# Prevalence, comorbidities, and profiles of neurodevelopmental disorders according to the DSM-5-TR in children aged 6 years old in a European region

**DOI:** 10.3389/fpsyt.2023.1260747

**Published:** 2023-11-10

**Authors:** Lorena Francés, Antoni Ruiz, C. Virgínia Soler, Joan Francés, Jessica Caules, Amaia Hervás, Carolina Carretero, Bárbara Cardona, Elizabeth Quezada, Alberto Fernández, Javier Quintero

**Affiliations:** ^1^Child and Adolescent Psychiatrist, IBSMIA, Universidad Complutense de Madrid, Madrid, Spain; ^2^Research Group on Socio-Educational Interventions in Childhood and Youth, University of Barcelona, Barcelona, Spain; ^3^Dalt Sant Joan Center, Mahón, Spain; ^4^Physical Activity and Sport Sciences, Miguel Hernández University, Elche, Spain; ^5^Teaching, Arrels Institute, Ciutadella de Menorca, Spain; ^6^Child–Adolescent Mental Health Unit, Mutua Terrasa University Hospital, Terrassa, Catalonia, Spain; ^7^Psychiatry, Autonomous University of Barcelona, Bellaterra, Catalonia, Spain; ^8^Saint George Hospital, London, United Kingdom; ^9^Child–Adolescent Psychiatry, Maudsley Hospital, London, United Kingdom; ^10^UCSMIA, UBS Es Mercadal, Menorca, Spain; ^11^UBS Es Castell, Menorca, Spain; ^12^CS Canal Salat, Ciutadella de Menorca, Spain; ^13^Psychiatry Department of Complutense University of Madrid, Madrid, Spain; ^14^Psychiatry Service of Infanta Leonor Hospital, Madrid, Spain

**Keywords:** neurodevelopmental disorders, prevalence, childhood, DSM-5-TR, ADHD, ASD, dyslexia, language disorders

## Abstract

**Background:**

There are no studies that measure the prevalence and real comorbidities of neurodevelopmental disorders (NDDs) according to the DSM-5-TR in 6-year-old children in population and clinical samples or studies that measure them as a whole. The data on the prevalence of these disorders are usually disparate because of the estimation methods (direct/indirect), the type of sample (population/clinical/school), and the ages studied.

**Methods:**

The initial sample (289 subjects) was representative of 6-year-old children in the entire population of Menorca, obtained from pediatric primary care services (100% of the sample). The patients were divided into two groups based on the criterion of verification of clinical warning signs. One of the groups represented the clinical or experimental sample (EG) (81 subjects) at risk of NDDs; the other group was considered the control sample (CG) (210 subjects), and they were subjects without risk of suffering NDDs. A direct clinical assessment of the clinical sample was carried out, and they were administered the *Wechsler Intelligence Scale for Children* (WISC-V), the *Clinical Evaluation of Language Fundamentals (CELF-5)*, the *Battery for the evaluation of the processes of revised reading (Batería para la evaluación de los procesos de lectura revisada – PROLEC-R)*, the *Test for the Diagnosis of Basic Mathematical Competences, (TEDI-MATH),* and the Developmental Coordination Disorder Questionnaire (DCDQ).

**Results:**

A total of 21.5% of the initial sample suffered from an NDD. A total of 2.4% presented autism spectrum disorder (ASD); 14% presented attention-deficit hyperactivity disorder (ADHD); 0.34% presented mild intellectual disability; 9.54% presented communication disorder (CD) (5.8% language disorder, 3.4% phonological disorder, and 0.34% stuttering); 10% presented learning disorder with reading difficulties; 5.8% presented learning disorder with difficulties in writing; 3.11% presented learning disorder with difficulties in mathematics; 1% presented transitory tic disorder; 0.34% presented chronic tic disorder; 1% presented Tourette syndrome; 2% presented motor coordination disorder (MCD); and 0.34% presented stereotypic movement disorders. Male children were more affected than female children in general, with male/female ORs of 0.14/0.92 for the presence of comorbidities, 0.11/0.88 for combined ADHD, 0.06/0.87 for language disorder, 1.02/1.27 for MCD, and 1.39/1.02 for inattentive ADHD.

**Conclusion:**

In disadvantaged contexts, there was a higher prevalence of NDDs and comorbidities, unless the disorder was extreme, in which case only the NDD manifestations were presented. A significant proportion of the sample had not been previously diagnosed (88.6%); therefore, early detection programs are recommended to identify warning signs and develop policies that help and support the most disadvantaged sectors of the population.

## Background

According to the latest revised version of the DSM-5 (1), neurodevelopmental disorders (NDDs) are a group of conditions that appear during the developmental period and usually manifest at an early stage, often before the child enters school. These deficits, although they improve with age, are generally related to functional interference in adult life. They are characterized by developmental deficits or differences in brain processes that produce alterations in personal, social, academic, or occupational functioning. The range of developmental deficits or differences varies from very specific limitations in learning or the control of executive functions to global deficits in social skills or intellectual capacity. Importantly, there has been a paradigm shift in the diagnosis of NDDs; in previous versions, NDDs were considered categorically defined, a fact that entailed differences in diagnosis and, as a consequence, in the prevalence obtained. In the new versions of both the ICD-11 (2) and DSM-5, dimensional approaches are considered to account for ranges of severity, often without a very clear boundary with neurodevelopment. Thus, the diagnosis of a disorder requires the presence of symptoms and functional alterations.

The NDD category includes disorders that manifest in a general way in almost all domains of development, such as intellectual disability (ID), as well as those that affect more specific domains, such as attention-deficit hyperactivity disorder (ADHD) and its three presentations (inattentive, hyperactive–impulsive, and combined); autism spectrum disorder (ASD); communication disorders (CDs), which include phonological disorders, language disorders and stuttering; specific learning disorders (including reading, writing, and mathematics); and motor disorders (tics, Tourette syndrome, motor coordination disorder, and stereotypic movement disorders).

NDDs often coexist with each other, and it is rare for them to occur alone. Homotypic comorbidity data are still scarce, and there are studies that investigate comorbidities within each disorder in particular, usually coinciding with those that are more present in the literature, such as autism (3, 4) or ADHD (5). It is unusual for comorbidities to be investigated in such studies, and there are also few studies that even consider them. However, our team studied and estimated comorbidity risk figures, with the most frequent combination being the presence of learning and language disorders, affecting 6.9% of the sample. The second most frequent combination was the presence of learning, language, and ADHD difficulties, affecting 4.5% of the sample (6). In Japan, the comorbidities among ADHD, ASD, and dyslexia were investigated (7). In Scotland (8), the most frequent comorbidities between ASD and ID were identified.

Prevalence and meta-analysis studies appear more frequently in scientific annals. The methods for estimating prevalence are sometimes unclear, thus potentially introducing bias (9). Depending on where the analyzed samples were recruited (i.e., a clinical, school, or population sample), very different figures are obtained. NDDs are considered underdiagnosed (10). In a previous systematic review by our research team (9), we found that the global prevalence rate of NDDs fluctuates globally between 4.70% in Scotland (8) to 55.5% in Norway (11) and 88.50% in Japan (7). In the United States, according to data published by the National Center for Health Statistics (NCHS) in 2015, an estimated 15% of children between the ages of 3 and 17 years are affected by NDDs (12). In a study carried out by our team and on a population sample, we found a global risk of presenting an NDD of 55.4% (6). An important fact to consider is that, intuitively, higher prevalence figures should coincide with more selected populations, that is, clinical samples, as is the case for the Norwegian study (11). However, in a population sample of 5-year-olds in Japan (13), estimated rates of ASD were similar to those for a clinical sample in Catalonia, covering a wider age range (2–17 years) (14). In addition, another fact that confirms the disparity in reported results is the low prevalence figures in a Spanish study carried out by pediatricians, in which the sample was selected based on follow-up in a child–adolescent psychiatry unit (15) and in which the age range was broad (0–14 years). Perhaps prevalence estimates would be more precise if studies employed more homogeneous criteria, such as narrower age ranges or similar or close ages not exceeding a 2-year margin, as is the case in this study. If samples were homogenized by narrower age ranges, the variability of results would decrease and results could be better compared. To be able to compare results, many studies would be needed in different populations (populations, schools, and clinics), with more homogeneous ages and similar evaluation methods (direct or indirect). In short, although these types of studies are on the rise, the number is still insufficient, and they yield mixed results.

In the literature reviewed in 2022 by our research team (9), the prevalence rates reported were as follows: ID, 0.63%; ADHD, 5–11%; ASD, 0.70–3%; specific learning disorders (SLDs), 3–10%; communication disorders (CDs), 1–3.42%; and motor disorders (MDs), 0.76–17% (3, 6–11). The estimated prevalence rates of the most common NDDs were as follows: ADHD, 7.9–9.5% (16, 17); ASD, 0.7–2.2% (16, 18, 19); SLDs (including developmental dyslexia [DD]), 1.2–24% (20, 21); and MDs, 1.4–19% (22, 23).

In our previous screening study, carried out through direct evaluations on children and parents (6), we established the following risks: a 23.4% risk of presenting ADHD in any of its modalities (inattentive, hyperactive–impulsive, and combined), a 2.8% risk of ASD, a 30.6% risk of presenting a learning disorder with reading difficulties, a 5.5% risk of tics, and a 22.5% risk of language problems (incomprehensible language or minor language problems).

The data on the prevalence of these disorders are usually disparate depending on the sample analyzed (clinical, school, or population) and the method used for estimating the prevalence (direct or indirect). In addition, the wide range of ages that are taken into account in each study in the scientific literature adds heterogeneity.

From a gender perspective, boys tend to be more affected by any NDD except inattentive ADHD, for which girls are more affected. In Scotland (8), the most frequent comorbidities were ASD and ID, occurring in 0.3% of children, 81.0% of whom were boys. Multimorbidity was prevalent with ASD and ID. ADHD, by itself or coexisting with other conditions, was the factor with the greatest weight in the increase in school exclusion. Multimorbidity was more common among boys, and the prevalence increased with school deprivation. In contrast, there was a greater negative impact on girls than boys. Regarding the NDDs studied and their comorbidities, 66.3% of the children included in a study by Hansen et al. (11) were boys, and Saito et al. (13) reported a male:female ratio of 2.2:1. Regarding ADHD, male:female ratios of 4:1 and 2:1 have been reported (24), coinciding in general with 3.2:1 reported by Sayal et al. (25) and Faraone et al. (26). Finally, in a study by Pérez-Crespo et al. (14), the male:female ratio was 4.5:1 for children with ASD. It is important to consider biological (genetic) and contextual risk factors (economic resources and educational level) to understand environmental factors as substantial epigenetic modulators. The factors that contribute to the increase in the number of male individuals with NDDs are complex and involve interactions among genetics, hormones, and environmental factors (27). Likewise, there are numerous studies that demonstrate the interference of prenatal and perinatal risk factors that modulate genetic expression in neurodevelopment (3, 28–32) and that investigate the influence of the environment (33, 34). Therefore, being a man and having low socioeconomic resources constitute a risk factor for suffering from one or more NDDs (6). It is important to recognize the general underdiagnosis of NDDs in female individuals and determine the reasons for this striking difference between male and female individuals. There is a long way to go to understand the reasons why the prevalence of NDDs is lower among female individuals than male individuals. One of the most relevant reasons is that research has focussed more on masculinity (35); other possible explanations could be the socially learned behaviors and the expected behavioral stereotypes for each gender. More research from different gender perspectives and studies that take into account gender differences in the expression of NDDs are needed.

The main objectives of this study were to determine the prevalence rates, comorbidities, and NDD profiles according to the latest version of the DSM-5-TR in a population of 6-year-old boys and girls who were followed longitudinally up to 8 years of age. In the data analysis, gender was taken into account, considering the different manifestations of symptoms and consequences for boys and girls.

The secondary objectives, derived from the prevalence data obtained, were to recommend resources to improve the early detection of these disorders and improve clinical care in the studied region.

It is important to promote research with direct methodologies through clinical interviews with children, parents, and teachers. As this was a study with direct evaluations, neuropsychological examinations were important in our study, and school information served as a very valuable tool for clinical diagnosis.

## Methods

**Figure 1:**
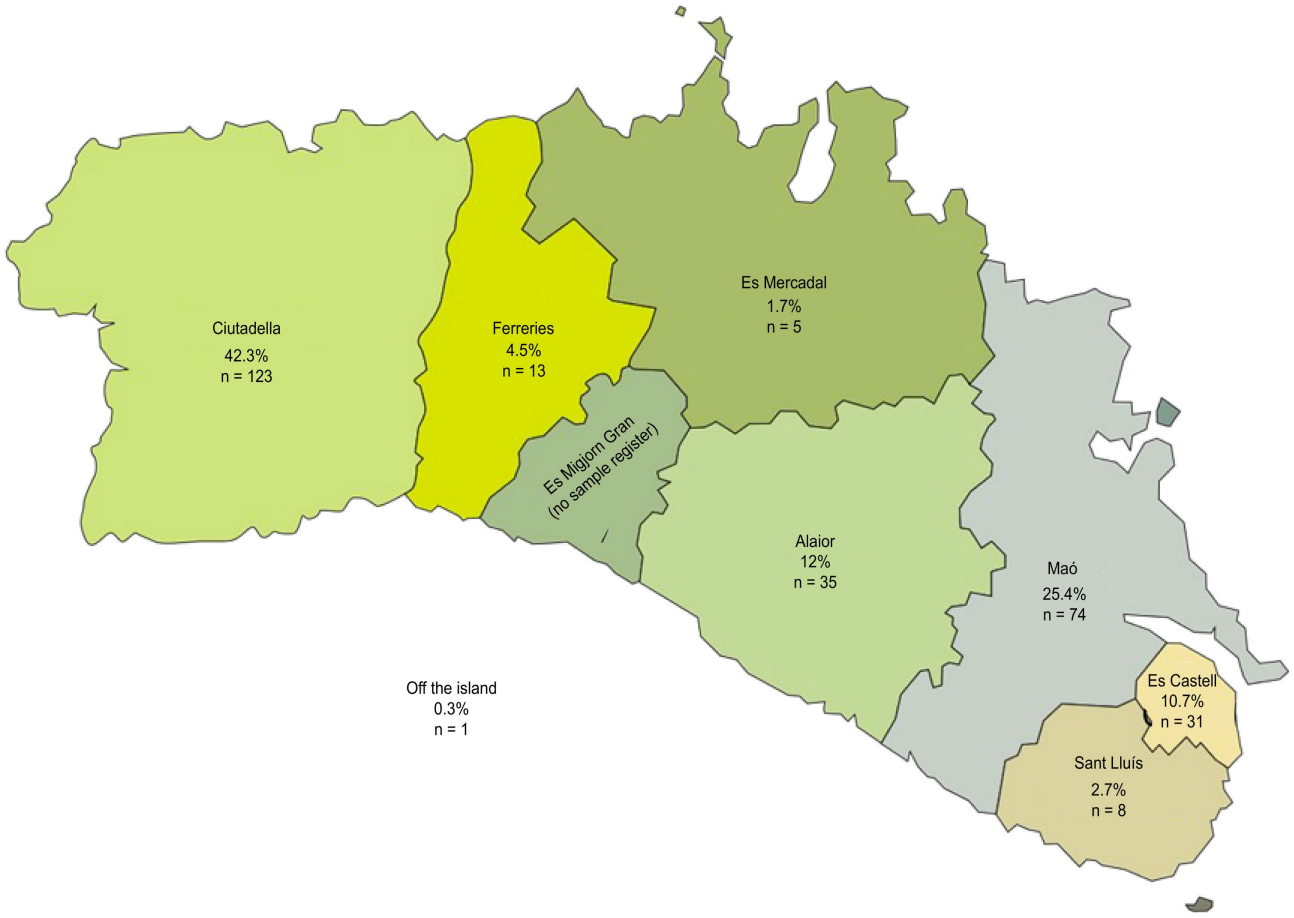
Map of the participating municipalities.

The objective of our study was to determine the prevalence of NDDs in children on the island of Menorca who attended a 6-year check-up at a pediatric primary care center affiliated with public health services, as per the child–adolescent program of the Balearic Islands (36), as well as to determine different profiles through multivariate analysis. A sample was recruited directly from the population registered and affiliated with the health centers of each municipality through consecutive opportunistic selection. The sample was recruited from the Menorcan population, and all the health centers of Menorca participated: the health center (Centro de Salud – SC) of Mahón (Dalt Sant Joan), CS Es Castell, CS Ferreries, CS Es Banyer, CS Mercadal, CS Sant Lluís, and Ciutadella (Canal Salat). Figure 1 shows the proportion of participation by municipality and the number of participating subjects. Collaboration rates were higher in Canal Salat (Ciutadella) (75%), CS Es Castell (70%), Dalt Sant Joan (Mahón) (50%), and Es Banyer (45%).

The sample size for an estimated maximum prevalence of NDDs of 25% on the island of Menorca to achieve a precision of ± 5% with a confidence interval of 95% and *p* = 0.25 was 289 subjects. The sample size was calculated using the 2021 registry (which refers to 1 January 21); the 5-year-old population (born in 2015) included 850 subjects, and the 6-year-old population (born in 2014) included 821 subjects. Therefore, to obtain a sample with adequate representativeness for this type of study (sampling error of 3 to 5%), 289 subjects were necessary. After receiving the approval of the Ethics Committee of the Balearic Islands (comité ético de las Islas Baleares—CEIB) in December 2020, the sample was recruited consecutively by pediatricians and nurses during the months of January, February, and March 2021, the time necessary to obtain a representative sample size of 289 children. Parents of children who attended the 6-year check-up were invited to participate in the study, and subjects who agreed to participate were included. The researcher and collaborators evaluated the parents who agreed to participate in the study after they had signed the informed consent form. At all times, security measures were taken to guarantee the confidentiality of the data. A total of 345 subjects were initially recruited through pediatricians. In this recruitment phase, 38 subjects were lost due to personal reasons and travel difficulties; therefore, 307 children were evaluated in the first phase of the study. Of these 307 participants, the sample was reduced to 289 (83.7% of the initial sample), with 18 losses due to incomplete evaluations, lack of information, and dropout.

This sample of 289 subjects (initial sample) was divided into a clinical or experimental group (EG) composed of those individuals who presented a risk of presenting NDDs measured through tests and/or clinical interviews. The EG was followed longitudinally to confirm all diagnoses, including learning disorders, and was carried out in the Community Mental Health Unit for Children and Adolescents (UCSMIA, for its acronym in Spanish) within the Balearic Institute for Childhood and Adolescent Mental Health (IBSMIA, for its acronym in Spanish).

The control group (CG) was composed of those individuals who did not present any risk of developing NDDs based on the results of the assessment and screening phase.

### Inclusion and exclusion criteria

Children who attended the 6-year-old consultations per the Infant-Youth Health Program at primary care centers in Menorca, which could be carried out from 2 months before reaching the age of 6 years up to 1 month before turning 7 years, were included.

Children diagnosed with NDDs at previous ages were not excluded and were included in the EG, and reports from accredited entities with specialized professionals were accepted.

All children younger than 5 years and 11 months and older than 7 years at the time of evaluation were excluded.

### Sociodemographic characteristics of the study population

The sample population was children affiliated with social security; therefore, 100% of the sample had data in the public health system database (Ib-salut). Importantly, 5% of the children had concomitant private and public monitoring, that is, they were affiliated with the social security system but also received care from private services, a common practice on the island. Data on ethnic and racial diversity were not collected. Of the included population obtained through consecutive random selection, 46.7% were girls (*n* = 136), and 53.3% were boys (*n* = 155). These children attended a total of 54 different schools on the island. The level of perceived economic resources was predominantly medium (89.7%), determined by a subjective evaluation completed by the parents about their perceived socioeconomic situation.

The study was carried out 1 year after the declaration of COVID-19 as a pandemic; all required safety measures were adopted, and masks were used during evaluations, facts that must be taken into account when interpreting the results.

### Study description

The sample was recruited from children attending routine child wellness consultations at primary care centers per the Child and Youth Health Program of the Balearic Islands (36). Subsequently, the families who decided to participate in the study were contacted by professionals who specialized in neurodevelopment and participated in an exhaustive general evaluation (of the child and family, separately) with different instruments that assessed different areas and warning signs. The clinical data were collected through a case report form that included risk factors associated with NDDs previously described in the literature, for example, prematurity, low birth weight, prenatal and perinatal infections, medical history, parental age and exposure to toxic substances, exposure to screens, type of diet, and participation in sports. Instruments that evaluate warning signs (Table 1) were used as screening tools to measure the risk of developing NDDs. The children were classified as having a risk or no risk of presenting NDD, yielding the experimental group (EG) and control group (CG), respectively. During the evaluation of the 289 subjects and their families, approximate times of 20–30 min and 30–40 min were required for the direct observation of the child and his/her parents, respectively.

**Table 1:** Instruments used for the direct assessment of subjects.

Test	Scope and features	Ages	Version and authors	Validity data
Wechsler Intelligence Scale for Children WISC-V (37)	IQ test administered individually. Measures global intelligence quotient and 15 tests organized in three different levels.	6 to 16 years and 11 months	WISC-V (37). The Spanish adaptation of the WISC-V was launched in 2015. As a strength, new measures of fluid reasoning, visuospatial, and working memory were added in this version.	There are few studies that confirm the replicability and validity of this scale. There are studies in which versions in different languages are validated. Data are lacking on the differential functioning of the items in relation to gender, age, or other variables of interest, the measurement invariance of the scores, and the information function under IRR.
Autism spectrum quotient (child version, AQ-Child)	Autistic traits. Questionnaire containing 50 questions answered by parents; a score greater than or equal to 75 points indicates a risk of ASD.	4–11 years	The Spanish version developed by the Autism Research Center (Cambridge) was used; this version is designed to be administered to parents. Authors: Auyeung et al. (38).	The psychometric validity and reliability of this instrument have not been reported in the scientific literature.
PROLEXIA battery for the early detection and differential diagnosis of dyslexia	Early detection of potential cases of dyslexia (Spanish).	4–6 years	Cuetos et al. (39).	Scarce (6), recently developed.
Revised battery for the evaluation of reading processes (PROLEC-R)	Evaluate reading (accuracy and speed) in Spanish.	6 to 12 years.	Cuetos et al. (40).	Most widely used assessment instrument for the Spanish language. Some studies have used it (41), and it has been validated in other languages (41).
Battery for the evaluation of writing processes (PROESC)	Writing processes.	8–15 years	Cuetos et al. (42).	Scarce.
Test for the Diagnosis of Basic Mathematical Competences (TEDI-MATH)	Evaluates the difficulties that children present in the numerical field.	4–8 years	Grégoire et al. (43) version adapted to Spanish by Manuel J. Sueiro and Jaime Pereña.	Used in research studies (44, 45).
Clinical Assessment of Language Fundamentals, CELF-5	Identification, diagnosis, and monitoring of language and communication disorders.	5 to 15 years	Wiig et al. (46) Spanish adaptation.	Used in research (47, 48).
Developmental Coordination Disorder Questionnaire	Parent questionnaire designed to detect coordination disorders.	5 to 15 years	Wilson and Crawford (49).	There are validity studies (50–53).
Mediterranean diet quality index, KIDMED	Brief questionnaire that assesses the quality of the Mediterranean diet.	Any age	Serra-Majern et al. (54).	Used in studies (54, 55).
Case report form	Used to collect data on sociodemographic variables, medical and mental health history of the mother and child, lifestyle habits, and general medical information; consists of 120 questions.	Any age	Our research team.	Not applicable.
Sally–Anne test	Explore the theory of mind through a brief history.	From 4 years	Baron-Cohen et al. (56).	Multitude of research on the theory of mind (57).
Child and Adolescent Assessment System (SENA, for its acronym in Spanish)	Detection of a wide spectrum of emotional and behavioral PROBLEMS.	3 to 18 years	Sánchez-Sánchez et al. (58).	Scarce, recently developed.
Behavior Rating Inventory of Executive Function (BRIEF-2)	Evaluation of executive functions by parents and teachers.	5–18 years	Gioia et al. (59). Spanish adaptation Belmonte et al. (60).	Used in studies (61).
Revised Perception of Differences Test (FACES-R)	Measures perceptual and attentional skills through 60 graphic items consisting of schematic drawings of faces with elementary lines.	6–18 years	Thurstone. Adaptador: Thurstone and Yela (62).	Recently developed, used to measure more than 12,000 Spanish schoolchildren.
Revised Children Sustained Attention Task (CSAT-R)	Version of the Continuous Performance Test (CPT) for the evaluation of sustained attention capacity in children.	6–11 years	Servera and Llabrés (63).	Scarce.
Neuropsychological Assessment of Executive Functions in Children (ENFEN, for its acronym in Spanish)	Maturity level assessment and cognitive performance in activities related to executive functions in children.	6–12 years	Portellano et al. (64).	Recent study (65).

During the assessments of children in the EG, a total of approximately 10 h was required for the neuropsychological examination, and a total of 6 h of clinical interviews was conducted with a child psychiatrist in the Child and Adolescent Mental Health Unit. Individuals with suspected cases of ASD and ADHD were discussed and supervised by different professionals within the existing protocols and working groups. Individuals with suspected ASD were evaluated with Autism Diagnostic Interview-Revised (ADI-R) and Autism Diagnosed Observation Schedule-Second Edition (ADOS-2). In addition, pertinent school reports were requested considering established territorial protocols. Individuals from whom there was clinical suspicion of an NDD were supervised by trained clinicians. Notably, the evaluations were conducted at different times to adhere to the following principles: do not spend more than 1 h per session to avoid distractions and use games to promote motivation and rest to avoid test fatigue. The schools that provided the required reports were informed. It is important to note that the clinical diagnoses followed the DSM-5-TR criteria (1).

### Measurements

Table 1 shows the measures used for the neuropsychological examination and the complete assessment.

The tests that were used were all adapted to the Spanish language, which was the language used to carry out the examinations.

### Data analysis

For the data analysis, a mixed methodology was used, and information exploration methods were combined through the use of standardized diagnostic tests and semistructured clinical interviews. The EG completed the WISC-5, CELF-5, PROLEC, PRO-ESCRI, TEDI-MATH, DCDQ, and a clinical assessment through interviews. Both groups (EG and CG) completed the KIDMED, the SENA, the PROLEXIA, the AQC, and the case report form. For the analysis of the data obtained, descriptive and inferential statistics were calculated, and “cluster” procedures were performed to obtain the profiles from a criterion variable.

The analysis was descriptive and quantitative in nature and included a univariate and/or bivariate analysis and a multivariate analysis. Specifically, for the descriptive analyses (univariate and bivariate), the percentages for each of the variables involved were calculated, and for the multivariate analysis, classification analysis with a criterion variable was used. SPSS (version 27) (66) was used for the univariate analysis, and SPAD (version 5.6) was used for the multivariate analysis (67). The latter allows profiles to be obtained from a variable that is to be characterized by the variables used in the tests administered in this study.

## Results

The variables used in the study and the prevalence results obtained for the initial population (*n* = 289) are presented below.

Subsequently, the two groups, i.e., experimental group (EG) and control group (CG), are compared.

Additionally, the profiles of the EG are described.

### Univariate analysis

The variables analyzed in the study are shown in Table 2.

**Table 2:** Study variables.

Variable	Measure: Dichotomous category
Global aspects, diagnosis of NDDs	
Comorbidity	Yes – No (1,0): risk identified in at least two tests
Presence of risk of an NDD	Yes – No (1,0): at least one test score indicates the presence of a disorder
Sociodemographic and clinical data	
Sex	
Female	Yes – No (1,0)
Male	
Course	
Infant (p5)	Yes – No (1,0)
Primary (1P)	
Territory	
Ciutadella	Yes – No (1,0)
Alajor-EsMerca-Ferre	
Mao	
EsCaste_S. Lluis	
Financial resources	
Low	Classification based on the perceptual responses of the parents. Yes – No (1,0)
Medium	
High	
Premature birth	Yes – No (1,0)
Breastfeeding	Yes – No (1,0)
Low birth weight	Yes – No (1,0): 1 = with 2,500 g or lower
Congenital infection (Question 50)	Yes – No (1,0)
Pregnant age > 45 years	Yes – No (1,0)
Parent age > 45 years	Yes – No (1,0)
Toxic substances in pregnancy	Yes – No (1,0) consumption of tobacco, alcohol, hashish
Eutocic delivery	Yes – No (1,0)
Instrumental delivery	Yes – No (1,0)
Cesarean delivery	Yes – No (1,0)
Diagnostic tests (categorized; Yes – No)	
PROLEXIA	Yes – No (1,0): 1 = Very high, high, and moderate scores grouped as risk of suffering from dyslexia
ADHD-MiniKid	Yes – No (1,0) 1 = 6 or more items with risk
AQC	Yes – No (1,0) 1 = score 75 or higher
TICS-Mini-Kid	Yes – No (1,0) 1 = presence of tics
No diagnosis	Yes – No (1,0 or 1,2) 1 = Presence
Mild ID	
ASD	
Total ADHD	
Combined ADHD	
Inattentive ADHD	
Hyperactive–impulsive ADHD	
Language disorder	
Phonological disorder	
Stuttering	
Learning, reading disorder	
Learning, writing disorder	
Learning, mathematics disorder	
Transient tics	
Chronic tics	
Tourette syndrome	
Stereotype disorder	
Motor coordination disorder	
Borderline total IQ	
HC	
Dyslexia	
LOW WISC	
MIDDLE WISC	
HIGH WISC	

NDDs, Neurodevelopmental disorders; PROLEXIA, PROLEXIA Battery for early detection and differential diagnosis of dyslexia; ADHD-Mini-Kid, Mini-Kid ADHD-Mini International Neuropsychiatric Interview for Children and Adolescents; AQC, autism spectrum quotient (Children’s version, AQ-Child); TICS-Mini-Kid, Mini-Kid TICS-Mini International Neuropsychiatric Interview for Children and Adolescents; ASD, Autism Spectrum Disorder; ADHD, attention-deficit hyperactivity disorder; HC, high capacity; WISC, Wechsler Intelligence Scale for Children (WISC-V).

### Initial sample

Of the initial sample, 21.5% suffered from NDDs. Of the 289 subjects included in the initial sample, that is, all individuals in the CG and EG, 2.4% presented ASD; 14% presented ADHD; 0.34% presented mild intellectual disability; 5.8% presented language disorders; 3.4% presented phonological disorder; 0.34% presented stuttering; 10% presented learning disorder with reading difficulties; 5.8% presented learning disorder with difficulties in writing; 3.11% presented learning disorder with difficulties in mathematics; 1% presented transitory tic disorder; 0.34% presented chronic tic disorder, 1% presented Tourette syndrome; 2% presented MCD; and 0.34% presented stereotypic movement disorders.

Table 3 provides the prevalence rates obtained for the initial sample and the experimental group (EG).

**Table 3:** Prevalence of NDDs in the experimental group (EG) and initial sample.

NDD (Yes)	Samples			
	Initial population sample (289 subjects)		Experimental group (81 subjects)	
	N (Unweighted %)	95% CI (Weighted %)	N (Unweighted %)	95% CI (Weighted %)
No diagnosis	227 (78.5)	0.73–0.83 (78)	19 (23.4)	0.14–0.34 (23)
Mild intellectual disability	3 (1)	0.00–0.03 (1)	3 (3.7)	0.008–0.10 (3)
ASD	7 (2.4)	0.01–0.04 (2)	7 (8.6)	0.03–0.17 (8)
Total ADHD	41 (14.2)	0.10–0.18 (14)	41 (50.4)	0.39–0.61 (50)
Combined ADHD	28 (9.7)	0.06–0.13 (9)	28 (34.6)	0.24–0.46 (34)
Inattentive ADHD	8 (2. 8)	0.01–0.05 (2)	8 (9.9)	0.04–0.18 (9)
Hyperactive–impulsive ADHD	5 (1.7)	0.006–0.04 (1.7)	5 (6.2)	0.02–0.13 (6)
Language disorder	17 (5.9)	0.03–0.09 (5)	17 (20.9)	0.12–0.31 (21)
Phonological disorder	10 (3.5)	0.01–0.06 (3)	10 (12.3)	0.06–0.21 (12)
Stuttering	1 (0.3)	0.00–0.01 (0.3)	1 (0.34)	0.00–0.06 (1)
Learning disorder with reading difficulties	26 (9)	0.06–0.12 (9)	26 (35.8)	0.22–0.43 (32)
Learning disorder with writing difficulties	17 (5.9)	0.03–0.09 (5)	17 (20.9)	0.12–0.31 (21)
Learning disorder with difficulties in mathematics	9 (3.1)	0.01–0.05 (3)	9 (11.1)	0.05–0.20 (11)
Transient tics	2 (0.7)	0.001–0.02 (0.7)	2 (2.5)	0.003–0.08 (2)
Chronic tics	1 (0.3)	0.00–0.01 (0.3)	1 (1.2)	0.00–0.06 (1)
Tourette syndrome	3 (1)	0.002–0.03 (1)	3 (3.7)	0.008–0.10 (3)
Stereotypic movement disorder	1 (0.3)	0.00–0.01 (0.3)	1 (1.2)	0.00–0.06 (1)
Motor coordination disorder	6 (2.1)	0.008–0.04 (2)	6 (7.4)	0.02–0.15 (7)
Borderline total IQ	4 (1.4)	0.004–0.03 (1)	4 (4.9)	0.01–0.12 (4)

Estimates and 95% CIs. 95% CI, 95% confidence interval; NDDs, neurodevelopmental disorders; ASD, Autism Spectrum Disorder; ADHD, attention-deficit hyperactivity disorder; IQ, intellectual quotient.

### Comorbidity in the clinical sample (EG)

Table 4 provides the comorbidity sequences for the disorders identified in the clinical sample (EG) and the corresponding frequencies.

**Table 4:** Sequence of diagnoses case by case (*n* = 81).

Diagnoses	Frequency	%
Inattentive ADHD/Math learning disorder/Borderline IQ (TIQ)	1	1.2
Mild ID/Hyperactive–Impulsive ADHD/Phonological disorder/Motor coordination	1	1.2
Mild ID	1	1.2
Mild ID/Language disorder	1	1.2
No Diagnosis	19	23.5
Phonological disorder/Learning disorder, reading/Learning disorder, writing/Motor coordination disorder	1	1.2
Phonological disorder/Motor coordination disorder/TIQ	1	1.2
Learning disorder, reading	4	4.9
Learning disorder, reading/Learning disorder, writing	1	1.2
Phonological disorder/Learning disorder, reading/Learning disorder, writing	1	1.2
Language disorder	1	1.2
Language disorder/Learning disorder, reading/Learning disorder, writing/Learning disorder, mathematics	4	4.9
Language disorder/Phonological disorder	1	1.2
Transient Tics	2	2.5
Combined ADHD/Learning disorder, reading/Learning disorder, writing/Learning disorder, mathematics	2	2.5
Combined ADHD/Language disorder/Phonological disorder/Learning disorder, reading/Learning disorder, writing/Learning disorder, mathematics	1	1.2
Combined ADHD/Language disorder/Learning disorder, reading/Learning disorder, writing/Borderline TIQ	1	1.2
Combined ADHD/Language disorder/Motor coordination disorder	1	1.2
Inattentive ADHD/Language disorder/Learning disorder, reading/Learning disorder, writing/Motor coordination disorder	1	1.2
Combined ADHD	8	9.9
Combined ADHD/Motor coordination	1	1.2
Combined ADHD/Learning disorder, reading/Learning disorder, writing	3	3.7
Combined ADHD/Learning disorder, reading	1	1.2
Combined ADHD/Phonological disorder	1	1.2
Combined ADHD/Language disorder	2	2.5
Combined ADHD/Chronic tics	1	1.2
Combined ADHD/Tourette syndrome	1	1.2
Combined ADHD/Stereotypic movement disorder	1	1.2
Hyperactive–impulsive ADHD	2	2.5
Hyperactive–impulsive ADHD/Learning disorder, reading/Tourette syndrome	1	1.2
Hyperactive–impulsive ADHD/Language disorder/Learning disorder, reading	1	1.2
Inattentive ADHD	2	2.5
Inattentive ADHD/Phonological disorder	1	1.2
Inattentive ADHD/Phonological disorder/Learning disorder, reading	1	1.2
Inattentive ADHD/Language disorder	1	1.2
Inattentive ADHD/Learning disorder, reading	1	1.2
ASD/cognitive precocity	2	2.5
ASD/Language disorder/Tourette syndrome	1	1.2
ASD/Combined ADHD/Learning disorder, reading/Learning disorder, writing/Learning disorder, mathematics	1	1.2
ASD/Combined ADHD/Language disorder/Borderline TIQ	1	1.2
ASD/Combined ADHD/Stuttering/Cognitive precociousness	1	1.2
ASD/Combined ADHD/Phonological disorder/Learning disorder, reading/Learning disorder, writing	1	1.2
Total	81	100

ADHD, attention-deficit hyperactivity disorder; IQ; intelligence quotient; ASD, autism spectrum disorder; TIQ, borderline IQ.

A total of 23.4% of the examined subjects did not receive any neurodevelopmental diagnosis (19 cases without diagnosis). Of this group, some received other heterotypic diagnoses, such as emotional distress reactive to different situations (20%), and other neurodevelopmental conditions, such as intellectual precocity (1%).

The remaining 76.5% received between 1 and 6 homotypic diagnoses. A total of 27.2% received one diagnosis; 18.5% received two diagnoses; 8.6% received three diagnoses; 16% received four diagnoses; 4.9% received five diagnoses; and 1.2% received six diagnoses.

A frequent association between ADHD and learning disorders with reading and writing difficulties was observed. The association between communication disorder and learning disorder was also frequent. Although the sample was small, for ASD, there was an association with language disorder and cognitive precocity (the latter is not a homotypic diagnosis). Finally, the existence of phonological disorder is striking.

### Multivariate analysis

#### Profile analysis

First, the results of the descriptive statistics are presented, and later, the characterization analysis of the selected variables (“cluster” with criterion variable) is shown, which, as indicated by Martí and Ruiz-Bueno (68), is about finding “the most explanatory set of individuals of the modalities of a qualitative variable” and taking into account that each group must be as homogeneous as possible among its members and as heterogeneous in relation to the others (67).

A value of *p* of <0.01 was used to identify the significant categories in the profiles.

#### Profiles according to the second phase variable

In this section, the profiles of the populations into which the sample was divided, i.e., the EG and the CG, are identified.

To identify these profiles, the presence of warning signs was taken into account.

Table 5 shows the categories of the variables that are characteristic of each profile.

**Table 5:** Characterization of the experimental group (EG) and control group (CG).

Variable	Characteristic category	Test value	Probability
Experimental group (EG), n = 81 (28.03%)			
Risk	Clinical sample	18.14	0.000
Mini-Kid ADHD	Yes	12.96	0.000
Comorbidities	Yes	8.67	0.000
Language disturbances	Yes	4.92	0.000
Risk in PROLEXIA	Yes	3.78	0.000
AQC	Risk (75 or more)	3.20	0.001
Financial resources	Low	2.54	0.005
Census municipality	Mahon	2.29	0.011
Parent 1 education level	Primary	2.09	0.018
Parent 2 education level	Primary	1.96	0.025
Sports	No	1.90	0.029
Control group (CG), n = 208 (71.97%)			
No risk	M_Poblacio	18.14	0.000
Mini-Kid ADHD	No	12.96	0.000
Comorbidities	X_No	8.67	0.000
Language disturbances	No	4.92	0.000
PROLEXIA risk	No	3.78	0.000
AQC	No risk/Lower	3.20	0.001
Financial resources	High	2.71	0.003
Sports	Yes	1.90	0.029
Census municipality	6_Es Castell	1.85	0.032
Parent 2 education level	Secondary	1.74	0.041

PROLEXIA, PROLEXIA Battery for early detection and differential diagnosis of dyslexia; ADHD-Mini-Kid, Mini-Kid ADHD-Mini International Neuropsychiatric Interview for Children and Adolescents; AQC, autism spectrum quotient (Children’s version, AQ-Child); ASD, autism spectrum disorder; ADHD, attention-deficit hyperactivity disorder.

In the EG, individuals tested positive in the Mini-Kid test for ADHD and tics presented homotypic comorbidities, had language problems in early childhood, presented risks in the PROLEXIA test and in the AQC test, had low economic resources, were from Mahón, had parents with a primary education level, and did not usually participate in sports.

In the CG, the individuals tested negative in the Mini-Kid test for ADHD and tics did not have associated comorbidities, did not have language problems in early childhood, did not present risks in the PROLEXIA test and the AQC, had parents with abundant economic resources, usually participated in sports, lived in Es Castell, and had parents with a secondary education level.

#### Profiles according to the second phase variables: EG and CG

The sample was analyzed considering gender differences, with male individuals being more affected than female individuals in general, except for inattentive ADHD. In the comparison between the groups (EG and CG), an important influence of context (socioeconomic level, parental studies, and sports) was evidenced in the presence of comorbidities and in disorders such as ADHD and language disorders.

### Analysis of the experimental (or clinical) group

#### Profiles based on sex

In this section, EG profiles were identified based on sex (male or female) (Table 6).

**Table 6:** Characterization of the EG by sex.

Variable	Characteristic category	Test value	Probability
Female group, *n* = 34 (41.98%)			
Sex	Female	9.95	0.000
Male	No	9.95	0.000
Female	Yes	9.95	0.000
Inattentive ADHD	Yes	2.39	0.008
Language alterations	No	2.38	0.009
Comorbidities	No	2.25	0.012
Critical items in the SENA	0	2.13	0.017
Mini-Kid tics	No	2.09	0.018
Language disorder	No	2.06	0.020
Combined ADHD	No	2.04	0.021
Comorbidities	No	1.94	0.026
Mother’s age	28 or less	1.89	0.030
Mini-Kid ADHD	Attention	1.87	0.031
Motor coordination disorder	No	1.84	0.033
Age_6y_6mon_to11months	Yes	1.65	0.050
Male group, n = 47 (58.02%)			
Sex	Male	9.95	0.000
Female	No	9.95	0.000
Male	Yes	9.95	0.000
Inattentive ADHD	No	2.39	0.008
Language alterations	Yes	2.38	0.009
Comorbidities	Yes	2.25	0.012
Mini-Kid tics	Yes	2.09	0.018
Critical items in the SENA	2	2.06	0.020
Language disorder	Yes	2.06	0.020
Combined ADHD	Yes	2.04	0.021
Mother’s age	29–35 years	2.02	0.022
Comorbidities	Yes	1.94	0.026
Motor coordination disorder	Yes	1.84	0.033
Age_6y_6mon_to 11 months	No	1.65	0.050

ADHD-Mini-Kid, Mini-Kid ADHD-Mini International Neuropsychiatric Interview for Children and Adolescents; AQC, autism spectrum quotient (Children’s version, AQ-Child); TICS-Mini-Kid, Mini-Kid TICS-Mini International Neuropsychiatric Interview for Children and Adolescents; ADHD, attention-deficit hyperactivity disorder; SENA, Child and Adolescent Assessment System (SENA, for its acronym in Spanish).

For female individuals, there was a predominance of inattentive ADHD, and in general, there were no alterations in language. There were no comorbidities or other disorders present (tics, motor coordination disorders (D), language disorder, and combined ADHD), and the age of the mother was usually 28 years or younger. For male individuals, there was a predominance of combined ADHD, language problems, presence of comorbidities, tics, language disorders, emotional problems (as detected using the SENA), and motor coordination disorder, and the mother’s age was usually in the range of 29–35 years.

#### Profiles based on sex

Table 7 details the proportion of diagnoses in the EG by sex; the statistical significance (chi-square test) is shown in bold.

**Table 7:** Diagnostic odds ratio by sex for the EG.

Diagnosis (Yes)	Sex						Odds ratio			
	Female (1)			Male (2)			Advantage reason: 1/2			
	N	%	CI (95%)	N	%	CI (95%)	Odds	CI (95%)	χ^2^ (gl = 1)	*p*-value
**Comorbidities**	**12**	**30**	**0.16–0.46**	**40**	**70**	**0.53–0.83**	**0.37**	**0.14–0.92**	**4.653**	**0.031**
No diagnosis	10	52.6	0.28–0.75	9	47.4	0.24–0.71	1.75	0.62–4.95	1.157	0.282 (n.s)
Mild ID	0	0	0.00–0.00	3	100	0.29–1.00	1.06	0.99–1.15	2.254	0.133 (n.s)
ASD	1	14.3	0.00–0.57	6	85.7	0.42–0.99	0.20	0.02–1.86	2.412	0.120 (n.s)
Total ADHD	16	39	0.24–0.55	25	61	0.44–0.78	0.78	0.03–1.89	0.297	0.586 (n.s)
**Combined ADHD**	**7**	**25**	**0.10–0.44**	**21**	**75**	**0.55–0.89**	**0.32**	**0.11–0.88**	**5.063**	**0.024**
**Inattentive ADHD**	**7**	**85.5**	**0.47–0.99**	**1**	**12.5**	**0.00–0.52**	**11.92**	**1.39–1.02**	**7.553**	**0.006**
Hyperactive–impulsive ADHD	2	40	0.05–0.85	3	60	0.14–0.94	0.91	0.14–5.80	0.009	0.926 (n.s)
**Language disorder**	**3**	**17.6**	**0.03–0.43**	**14**	**82.4**	**0.56–0.96**	**0.22**	**0.06–0.87**	**5.228**	**0.022**
Phonological disorder	4	40	0.12–0.73	6	60	0.26–0.87	0.91	0.23–3.51	0.018	0.892 (n.s)
Stuttering	0	0	0.00–0.97	1	100	0.02–1.00	1.02	0.98–1.06	0.732	0.392 (n.s)
Learning disorder, reading	13	50	0.29–0.70	13	50	0.29–0.70	1.61	0.63–4.15	1.012	0.314 (n.s)
Learning disorder, writing	7	41.2	0.18–0.67	10	58.8	0.32–0.81	0.95	0.32–2.84	0.006	0.940 (n.s.)
Learning disorder, mathematics	4	44.4	0.17–0.78	5	55.6	0.21–0.86	1.12	0.27–4.52	0.025	0.874 (n.s.)
Transient tics	0	0	0.00–0.00	2	100	0.15–1.00	1.04	0.98–1.10	1.483	0.223 (n.s.)
Chronic tics	0	0	0.00–0.97	1	100	0.25–1.00	1.02	0.98–1.06	0.732	0.392 (n.s.)
Tourette syndrome	0	0	0.00–0.70	3	100	0.29–1.00	1.06	0.99–1.15	2.254	0.133 (n.s)
Stereotypic movement disorder	0	0	0.00–0.97	1	100	0.25–1.00	1.02	0.98–1.06	0.732	0.392 (n.s)
**Motor coordination disorder**	**0**	**0**	**0.00–0.45**	**6**	**100**	**0.54–1.00**	**1.14**	**1.02–1.27**	**4.688**	**0.030**
Borderline TIQ	1	25	0.00–0.80	3	75	0.19–0.99	0.44	0.44–4.46	0.498	0.480

95% CI, confidence interval; ADHD, attention-deficit hyperactivity disorder; IQ, intelligence quotient; ASD, autism spectrum disorder; TIQ, borderline IQ. Binomial success rate for a sample (Clopper–Pearson). The statistical significance (chi-square test) is shown in bold.

#### Profiles based on the WISC level

In this section, profiles are identified based on the WISC level. To identify these profiles, scalar variables were converted into categorical variables, following the criterion of the quartile score. The values have been grouped as follows: LOW WISC, total WISC scores lower than 89; MIDDLE WISC, total WISC scores within 90–109; HIGH WISC, total WISC scores greater than 110; and DK – NA WISC, no WISC score.

As seen in Table 8, individuals with a low WISC level had higher PROLEXIA test scores, altered CELF-5 test results, comorbidities, alterations in the development of language before 3 years, and borderline intelligence levels.

**Table 8:** Characterization of EG by WISC-5 level.

Variable	Characteristic category	Test value	Probability
Low WISC group, *n* = 32 (39.51%)			
WISC category	Low	9.87	0.000
WISC category	Normal-Low	6.96	0.000
WISC category	Lower	3.98	0.000
School	La Salle (Mahón)	2.20	0.014
PROLEXIA risk	Very high	2.17	0.015
CELF-5 results	Low	2.08	0.019
Borderline TIQ	Yes	2.02	0.022
Comorbidities	Yes	1.89	0.029
Normal language development	No	1.75	0.040
Comorbidities	Yes	1.68	0.046
Middle WISC group, n = 30 (37.04%)			
WISC category	Normal-Medium	9.78	0.000
Wisc_3categories	Medium	9.78	0.000
Optimal kidmed	No	2.81	0.002
Kidmed category	Improve pattern	2.57	0.005
Grouped age	6 years to 6 months	2.23	0.013
Age_6y_6mon_to 11 months	No	1.93	0.027
School	CEIP Pere Casanovas	1.67	0.048
High WISC group, n = 11 (13.58%)			
WISC category	High	7.37	0.000
WISC category	Normal-High	5.73	0.000
WISC category	Superior	2.89	0.002
PROLEXIA risk	Very low	2.78	0.003
Cross laterality	Yes	2.45	0.007
Mini-Kid ADHD	H-I	2.22	0.013
Breastfeeding	Yes	2.16	0.015
Breastfeeding months	16–25 months	2.13	0.016
Financial resources	Medium-High	1.91	0.028
Financial resources	High	1.71	0.044
Parent 2 education level	University	1.67	0.047

WISC, Wechsler Intelligence Scale for Children (WISC-V); PROLEXIA, PROLEXIA Battery for early detection and differential diagnosis of dyslexia; CELF-5, Clinical Evaluation of Language Fundamentals; ADHD-Mini-Kid, Mini-Kid ADHD-Mini International Neuropsychiatric Interview for Children and Adolescents; KIDMED, Mediterranean diet quality index; TIQ, borderline intelligence quotient.

Individuals with a high WISC level had very low risk (PROLEXIA test), crossed laterality, positive Mini-Kid ADHD HYPERACTIVE–IMPULSIVE and COMBINED, prolonged breastfeeding (between 21 and 25 months), medium-high economic resources, and parents with university studies.

#### Profiles based on high PROLEXIA results

Table 9 shows the categories of the variables that are characteristic of the profiles analyzed.

**Table 9:** Characterization of the EG by high PROLEXIA results.

Variable	Characteristic category	Test value	Probability
Yes group, *n* = 39 (48.15%)			
PROLEXIA high	Yes	10.04	0.000
Comorbidities	Yes	6.76	0.000
Learning disorder, reading	Yes	6.09	0.000
PROLEXIA risk	Very high	5.75	0.000
Language alterations	Yes	4.90	0.000
Diagnostics	3 or + diagnoses	4.72	0.000
Learning disorder, writing	Yes	4.21	0.000
No diagnosis	No	3.64	0.000
Language disorder	Yes	3.57	0.000
CELF-5 result	Low	2.83	0.002
WISC category	Low	2.79	0.003
Number of diagnoses	4	2.63	0.004
Sports	No	2.56	0.005
Normal language development	No	2.37	0.009
Learning disorder, mathematics	Yes	2.30	0.011
Drug exposure during pregnancy	Yes	2.01	0.022
PROLEXIA risk	Moderate	2.01	0.022
High WISC	No	1.85	0.032
WISC category	Lower	1.83	0.033
Mini-Kid ADHD	No	1.76	0.039
CELF-5 result	Medium	1.65	0.049
Number of diagnoses	5	1.65	0.049
No group, n = 42 (51.85%)			
PROLEXIA high	No	10.04	0.000
Comorbidities	No	6.76	0.000
PROLEXIA risk	Very low	6.11	0.000
Learning disorder, reading	No	6.09	0.000
Language alterations	No	4.90	0.000
PROLEXIA risk	Low	4.61	0.000
Learning disorder, writing	No	4.21	0.000
CELF-5 result	Medium	3.77	0.000
Diagnostics	Without	3.64	0.000
Number of diagnoses	0	3.64	0.000
Language disorder	No	3.57	0.000
Low WISC	No	2.79	0.003
Sports	Yes	2.56	0.005
Normal language development	Yes	2.37	0.009
Learning disorder mathematics	No	2.30	0.011
Drug exposure during pregnancy	No	2.30	0.011
Type of delivery	Instrumental	2.10	0.018
Mini-Kid ADHD	H-I	1.99	0.023
WISC category	High	1.85	0.032

PROLEXIA, PROLEXIA Battery for early detection and differential diagnosis of dyslexia; CELF-5, Clinical Evaluation of Language Fundamentals; ADHD-Mini-Kid, Mini-Kid ADHD-Mini International Neuropsychiatric Interview for Children and Adolescents; AQC, autism spectrum quotient (Children’s version, AQ-Child); TICS-Mini-Kid, Mini-Kid TICS-Mini International Neuropsychiatric Interview for Children and Adolescents; WISC, Wechsler Intelligence Scale for Children (WISC-V).

The group with high PROLEXIA results had comorbidities, presented risks (PROLEXIA), had a learning disorder with difficulties in reading, had language problems, presented three or more comorbid homotypic diagnoses, had a language disorder, had low CELF test results, had low WISC test results, used drugs during pregnancy, and did not usually play sports.

The group that did not have high PROLEXIA results did not present comorbidities, did not present risks (PROLEXIA), did not have language problems or other associated disorders, did not usually have associated homotypic diagnoses, had low WISC levels, did not use drugs during pregnancy, and usually participated in sports.

As seen, the education level of the parents and financial resources did not have much of an influence.

#### Profiles based on comorbidities

In this section, profiles are identified by the presence of comorbidities (Table 10).

**Table 10:** Characterization of EG profiles by comorbidities.

Variable	Characteristic category	Test value	Probability
Yes group, *n* = 40 (49.38%)			
Comorbidities	Yes	10.05	0.000
Diagnostics	3 or + diagnoses	6.40	0.000
No diagnosis	No	5.11	0.000
PROLEXIA high	Yes	4.66	0.000
Diagnostics	2	4.42	0.000
Language alterations	Yes	4.21	0.000
ADHD	Yes	4.19	0.000
Learning disorder, writing	Yes	4.10	0.000
Language disorder	Yes	4.10	0.000
Number of diagnoses	4	4.00	0.000
PROLEXIA risk	Very high	3.73	0.000
Learning disorder, reading	Yes	3.72	0.000
Learning disorder, mathematics	Yes	3.08	0.001
Normal language development	No	2.98	0.001
Combined ADHD	Yes	2.67	0.004
Number of diagnoses	3	2.55	0.005
Phonological disorder	Yes	2.49	0.006
Motor coordination disorder	Yes	2.26	0.012
CELF-5 result	Low	2.02	0.022
DCDQ result	Altered	1.95	0.026
Inattentive ADHD	Yes	1.94	0.026
Male	Yes	1.94	0.026
Female	No	1.94	0.026
SENA critical items	1	1.78	0.037
Sally–Anne test answer	Box	1.69	0.046
Low WISC	Yes	1.68	0.046
No group, n = 41 (50.62%)			
Comorbidities	No	10.05	0.000
Number of diagnoses	1	5.68	0.000
No diagnosis	Yes	5.11	0.000
Number of diagnoses	0	5.11	0.000
PROLEXIA high	No	4.66	0.000
Language alterations	No	4.21	0.000
ADHD	No	4.19	0.000
Learning disorder, writing	No	4.10	0.000
Language disorder	No	4.10	0.000
PROLEXIA risk	Very low	3.88	0.000
Learning disorder, reading	No	3.72	0.000
Learning disorder, mathematics	No	3.08	0.001
CELF-5 result	Medium	3.00	0.001
Normal language development	Yes	2.98	0.001
Combined ADHD	No	2.67	0.004
Phonological disorder	No	2.49	0.006
Motor coordination disorder	No	2.26	0.012
DCDQ	Normal	2.26	0.012
Biological father’s age	31 or less	2.13	0.016
Mother’s age	28 or less	1.98	0.024
Inattentive ADHD	No	1.94	0.026
Male	No	1.94	0.026
Sex	Female	1.94	0.026
Female	Yes	1.94	0.026
Sally–Anne test answer	No	1.69	0.046
Low WISC	No	1.68	0.046

PROLEXIA, PROLEXIA Battery for early detection and differential diagnosis of dyslexia; CELF-5, Clinical Evaluation of Language Fundamentals; ADHD-Mini-Kid, Mini-Kid ADHD-Mini International Neuropsychiatric Interview for Children and Adolescents; AQC, autism spectrum quotient (Children’s version, AQ-Child); TICS-Mini-Kid, Mini-Kid TICS-Mini International Neuropsychiatric Interview for Children and Adolescents; WISC, Wechsler Intelligence Scale for Children (WISC-V); ADHD, attention-deficit hyperactivity disorder; DCDQ, Developmental Coordination Disorder Questionnaire.

Individuals with comorbidities had 2, 3, and 4 comorbid diagnoses, had alterations in language in early childhood, had learning disorders with difficulties in reading, writing, and mathematics, had combined and inattentive ADHD, had motor coordination disorder, had language disorder, had phonological disorder, had emotional alterations, as determined using the SENA, had erroneous Sally–Anne test results, had a low WISC level, and were male individuals.

Individuals with no comorbid disorders, without comorbidities, had no risk (PROLEXIA), did not have language alterations, nor phonological disorder, language disorder, or motor coordination disorder, did not have abnormal CELF-5 and DCDQ results, had correct Sally–Anne test answers, and had high or mid-range WISC levels.

#### Profiles as a function of participation in sports

In this section, EG profiles are identified based on those who did and did not participate in sports (Table 11).

**Table 11:** Characterization of EG profiles by participation in sports.

Variable	Characteristic category	Test value	Probability
Yes group, *n* = 50 (61.73%)			
Sports	Yes	9.83	0.000
Financial resources	High	2.74	0.003
PROLEXIA high	No	2.56	0.005
Financial resources	Medium-High	2.40	0.008
Type of delivery	Eutocic	2.39	0.008
Language disorder	No	2.22	0.013
CELF-5 result	Medium	2.19	0.014
Comorbidities	No	2.15	0.016
Borderline TIQ	No	2.08	0.019
PROLEXIA risk	Very low	2.05	0.020
Motor coordination disorder	No	1.90	0.028
Language alterations	No	1.89	0.029
Mini-Kid ADHD	HI	1.88	0.030
Normal language development	Yes	1.85	0.032
Inattentive ADHD	No	1.85	0.032
Difficulty tying shoelaces	No	1.70	0.044
Repetitive play	Yes	1.65	0.049
No group, *n* = 31 (38.27%)			
Sports	No	9.83	0.000
PROLEXIA risk	High	2.56	0.005
Financial resources	Low	2.50	0.006
PROLEXIA risk	Very high	2.31	0.010
Language disorder	Yes	2.22	0.013
Comorbidities	Yes	2.15	0.016
Borderline TIQ	Yes	2.08	0.019
Delivery type	Cesarean section	2.01	0.022
Motor coordination disorder	Yes	1.90	0.028
Language alterations	Yes	1.89	0.029
Normal language development	No	1.85	0.032
Inattentive ADHD	Yes	1.85	0.032
Repetitive play	No	1.65	0.049

PROLEXIA, PROLEXIA Battery for early detection and differential diagnosis of dyslexia; CELF-5, Clinical Evaluation of Language Fundamentals; ADHD-Mini-Kid, Mini-Kid ADHD-Mini International Neuropsychiatric Interview for Children and Adolescents; KIDMED, Mediterranean diet quality index; TIQ, borderline intelligence quotient; ADHD, attention-deficit hyperactivity disorder.

Individuals who did participate in sports came from families with high or medium-high economic resources, did not present risk (PROLEXIA), did not have associated disorders or language alterations, did not have low WISC levels, did not participate in repetitive play, and had a eutocic delivery.

Individuals who did not play sports had a low income, had very high risk (PROLEXIA), had language disorder and language development problems, had comorbidities, had ADHD with inattentive presentation, presented a borderline total intellectual coefficient, had motor coordination disorder, did not participate in repetitive play, and were born by cesarean section.

#### Profiles based on the use of screens

In this section, profiles are identified based on the use of screens.

Individuals who used screens presented with combined ADHD and had an instrumental delivery.

Individuals who did not use screens had psychotic disorders in the paternal branch, had parents with a university education, were delivered by cesarean section, were usually from CEIP Tramuntana (school in contact with nature), and had correct Sally–Anne test responses.

#### Profiles based on the AQC test

The profile based on the AQC test suggests that individuals with results greater than or equal to 75 had a diagnosis of ASD.

## Discussion

The difference found in this study between the global prevalence of having one or more NDD, estimated at 21.5%, and the figure calculated in the initial screening, 55.4%, is striking (6). This difference could indicate non-diagnosis in screening tests, potentially due to an over detection by some screening tests, which can be highly polarized and only detect very high risks, to the speed with which some tests are performed in relation to other tests, and to the detailed *a posteriori* examinations performed on subjects in clinical samples. Regarding the possible over detection by screening tests, it is preferable to detect more risks than to miss some of them because it is important to prevent as many cases as possible and avoid false-negative cases. In short, for screening programs, tests should be simple to apply, accepted by patients or the general population, have minimal adverse effects, and be financially supportable (69). These aspects of sensitivity and specificity could not be estimated due to the limitations of the study design. However, in screening programs, it is important to have highly sensitive tests that avoid false negatives. In our study, we consider that the PROLEXIA test, Mini-Kid ADHD, and AQC could be quite sensitive and easily applicable screening tools due to their rapidity. We have observed that the PROLEXIA is a powerful test for detecting comorbidity.

As shown in Table 3, when comparing the prevalence rates for the EG with those for the initial sample, the rates are higher in the EG. Likewise, the percentage of subjects who did not receive a diagnosis was higher in the population sample (78.5%) than in the EG (23.4%).

One of the strengths of our study is the direct assessment of each individual both in the screening phase and in the diagnostic phase, using the neuropsychological examination as a tool to provide information to the clinician to make a diagnosis with the greatest precision and information possible, avoiding errors associated with estimates or rapid and indirect tests. In addition to direct evaluations, supervision of each patient was carried out by a team of professionals trained in neurodevelopment, and the evaluations by teachers and the children’s parents were taken into account.

The prevalence rates for each NDD in this study are similar to estimates reported in the literature. These figures are consistent regardless of age, which in our case were 6-year-old children, with associated cons (age limitation) and pros (large sample of subjects of the same age). The fact that studies present wide age ranges can lead to biases in the sense of identifying cases at an early age, which are of interest to us because early identification favors an early diagnosis of subtle warning signs. The age chosen for this study should be discussed. From the age of 6 years, clinicians diagnose almost all NDDs, except learning disorders. In this study, a longitudinal follow-up was performed that began at 6 years and ended at 8 years with the assessment of the learning sphere to confirm diagnoses. In this way, we were able to confirm diagnoses of those individuals at risk of suffering from learning disorders identified in the screening phase, i.e., when the children were 6 years old. With regard to ASD, 6 years is not an age of early diagnosis; however, our objective was to detect more subtle cases that could go unnoticed at an early age, e.g., cases of autism associated with a superior IQ (of which two cases were detected) or female autism. Both types tend to go unnoticed and are diagnosed in adulthood. It is likely that there is an underreporting of women with ASD, especially among those with high cognitive performance, an effect possibly related to the assessment techniques used. In general, women with autism have better early language development and better social skills, and their play may even develop as expected (70). We did not detect cases of severe ASD or moderate or severe ID; if there were any, they would have already been diagnosed at an earlier age.

The sample size (*n* = 289) could be considered a limitation of this study if compared with that in other population prevalence studies from other territories; however, it should be taken into account that this n is representative of the Menorcan population and that, assessing subjects in two periods with direct evaluations, it would be unfeasible and extremely expensive in terms of time spent, money, and personnel needed. In addition, there are no studies that cover specific ages but rather broader age ranges.

We observed that inattentive ADHD was predominant in women and that combined and hyperactive–impulsive ADHD was predominant in men, who were also more affected by the rest of the disorders, such as language disorder, motor disorder, ASD, and learning disorder. In addition, male sex is associated with higher rates of comorbidity.

Some findings of the multivariate analysis are notable. Being a girl is associated with younger mothers. The profile of children with a higher total intelligence quotient (IQ) was more likely to have hyperactive–impulsive ADHD (potentially because they are more curious), and a high TIQ was associated with longer breastfeeding (between 15 and 25 months). The authors of previous studies concluded that “breastfeeding could significantly improve the intelligence of children, with a duration >6 months result in an intelligence score slightly but significantly higher than that for a duration ≤6 months” (71) and that breastfeeding is related to higher performance on intelligence tests (72, 73). Furthermore, the authors of a previous review (74) concluded that “breastfeeding has a small positive effect on IQ in late childhood. The evidence to suggest that breastfeeding is a protective factor in the development of conduct disorders and the achievement of greater executive function is limited.” All these could be influenced by context, that is, higher educational and socioeconomic levels. In the profiling, we only found that an “optimal diet” determined by KIDMED is characteristic of individuals with an average IQ determined by WISC. This is the reason why we have not gone on to make profiles based on diet, since it seems that the variables sports and screen use appear more frequently in the characterization of profiles. In this sense, sports and less exposure to screens could be linked to less comorbidity.

Disadvantaged contexts in the development of disorders such as language disorders and ADHD are more independent of genetic factors. As seen in learning disorders, the educational level of the parents and financial resources does not influence learning disorders as much as other disorders (35, 75, 76).

In this study, the environment is an island with a population that has tended to remain stable over time, indicating that the results would be more consistent with reality, i.e., with little change. However, the environment of the island could be a limitation; it is a semirural area, where the largest urban centers are two small cities that do not exceed 30,000 inhabitants. This should be taken into account when comparing the results herein with those of studies where the population is more changeable and where population centers tend to be more diverse.

The analyses used were selected on the basis of the intentions of the study, that is, a descriptive and exploratory focus on the EG. Bivariate analyses were used to contrast population-type samples and sociodemographic aspects, more specifically gender, due to its importance as indicated by the latest evidence for each disorder (27, 77, 78). As the EG had a small sample, adequate statistical inferences are difficult because it was not possible to use parametric tests; for this reason, cluster analysis by classification (classification analysis with criterion variable) was used.

In this study, a population sample that could have clinical manifestations was analyzed. The sample size could have been increased by recruiting from schools; it is unknown how this approach would have influenced the representativeness of the results obtained. The method chosen is consistent with the goal of promoting early detection at primary care centers and facilitating communication between primary and specialized services, which is scarce and necessary in our environment.

## Conclusion

The importance of this study is the direct assessment of each individual in the sample through screening tests and clinical interviews and the use of a neuropsychological examination as a complementary diagnostic tool that, in many mental health centers, continues to be difficult to apply due to time limitations for consultations. The multidisciplinary work that has been carried out throughout the study’s trajectory is noteworthy, starting with primary care pediatric services to specialized mental health units, schools, and a neuropsychology team with extensive experience. One limitation of the public health system is the lack of time and professionals for the exhaustive assessment of each disorder. There can be doubts regarding the diagnosis of certain disorders when there is a lack of neuropsychological and human tools to perform assessments, which is the case for language and learning disorders in our community, a fact that delays diagnoses and results in higher rates of school failure (76, 79).

The results for the sample appear to be consistent with those reported in the scientific literature and with the predictions of clinicians.

The comorbidity of ADHD and learning disorder was observed, as also evidenced in scientific annals (76), as was general multimorbidity in male individuals (35, 80) and a predominance of inattentive ADHD in female individuals. The prevalence of communication disorder, both language disorder and phonological disorder, and its association with learning disorder and ADHD is apparent. Future research should study the nature of these associations.

In disadvantaged contexts (low economic resources and lower levels of education of parents), there is a higher prevalence of NDDs, except if the disorder is very extreme or genetically determined (such as dyslexia or ASD), which will manifest regardless of the environment. An important influence of context has been evidenced in the presentation of comorbidities in language disorders and ADHD.

Sociodemographic variables could be as powerful predictors as screening tests, or having a sociodemographic variable from a disadvantaged context should alert clinicians to the possibility of an NDD. Profiles of the disadvantaged context include low economic resources, lower levels of education of parents, and lifestyle habits that can be improved.

Additionally, policies should be implemented that provide public services with tools and personnel to be able to detect, diagnose, and treat NDDs, with an emphasis on learning and language difficulties, which are not easily detected because of a lack of resources.

In addition, we urge the development of health promotion programs in schools and CSs (balanced diet and physical activity) so that they can be extended to families and, therefore, societies. As Farholm and Sørensen (81) note, the modification of lifestyle factors, such as increased physical activity and improved diet, is associated with a reduction in health problems as well as improvements in mental health. Zaman et al. (82) affirm that positively modifying lifestyle factors, with an emphasis on food, diet, and exercise, can help to improve and/or prevent medical and psychiatric disorders. Exercise can be a marker of those populations with better socioeconomic conditions and greater cognitive abilities of parents who choose better lifestyle habits; however, it could not be ruled out that exercise *per se* is a factor associated with better neurodevelopment because it is a factor in very early brain development and implicated in many neurodevelopmental disorders.

Notably, a significant proportion of the sample had never been diagnosed (88.6%); for this reason, early detection programs are recommended that include psychoeducation for parents and the detection of warning signs by primary care services and schools. In short, policies are needed that help and support the most disadvantaged sectors of the population: The more socioeconomic resources in a population, the less risk it will have. In conclusion, context takes on a role almost as important as genetics or gender, with the difference that it can be modified. In this way, focusing on secondary prevention, risks can be reduced by improving contexts.

Context and the epigenetic modification that it exerts when accelerating the manifestation and presentation of NDDs can be evidenced in exposure to screens (and its association with higher rates of ADHD diagnosis as well as greater emotional dysregulation), a reduction in the risk of NDDs through participating in sports (6), and the vulnerability of individuals in the most disadvantaged contexts, i.e., low income and lower education level of parents (in language disorder and ADHD, it exerts accelerating by exposure to screens). For this reason, it is of paramount importance in view of health planning to support and invest in policies that support these most affected sectors to prevent and reduce risk factors for NDDs.

## Data availability statement

The original contributions presented in the study are included in the article/supplementary material, further inquiries can be directed to the corresponding author.

## Ethics statement

The studies involving humans were approved by Comité de ética de les Illes Balears (CEIB). The studies were conducted in accordance with the local legislation and institutional requirements. Written informed consent for participation in this study was provided by the participants’ legal guardians/next of kin.

## Author contributions

LF: Conceptualization, Data curation, Formal analysis, Funding acquisition, Investigation, Methodology, Project administration, Resources, Software, Supervision, Validation, Visualization, Writing – original draft, Writing – review & editing. AR: Data curation, Formal analysis, Investigation, Methodology, Software, Writing – original draft. CS: Funding acquisition, Investigation, Resources, Writing – original draft. JF: Writing – review & editing. JC: Funding acquisition, Investigation, Resources, Writing – original draft. AH: Writing – review & editing. CC: Investigation, Writing – original draft. BC: Investigation, Writing – original draft. EQ: Investigation, Writing – review & editing. AF: Supervision, Writing – review & editing. JQ: Supervision, Writing – review & editing.

## Glossary

NDD: Neurodevelopmental disorder
ID: Intellectual disability
ADHD: Attention-deficit hyperactivity disorder
ASD: Autism spectrum disorder
SLD: Specific learning disorder (for example, dyslexia)
CD: Communication disorder
MD: Motor disorder
TS: Tourette syndrome
TD: Tic disorder
DCD: Developmental coordination disorder
DD: Developmental dyslexia
DLD: Development language disorder
SLI: Specific language impairment
DSM-5: Diagnostic and Statistical Manual of Mental Disorders, 5th Edition
ICD-10: International Statistical Classification of Diseases and Related Health Problems, 10th revision
ICD-11: International Statistical Classification of Diseases and Related Health Problems, 11th revision
WHO: World Health Organization
APA: American Psychiatric Association
